# Supplementary material for: PCGF5 is required for neural differentiation of embryonic stem cells
Source: Nat Commun. 2018 May 15;9:1463. doi: 10.1038/s41467-018-03781-0 (PMC5954019; doi:10.1038/s41467-018-03781-0)
Supplement: Supplementary file 1 — Supplementary Information [file 41467_2018_3781_MOESM1_ESM.pdf]

Supplementary Information

PCGF5 is required for neural differentiation of embryonic stem cells

Yao et al.

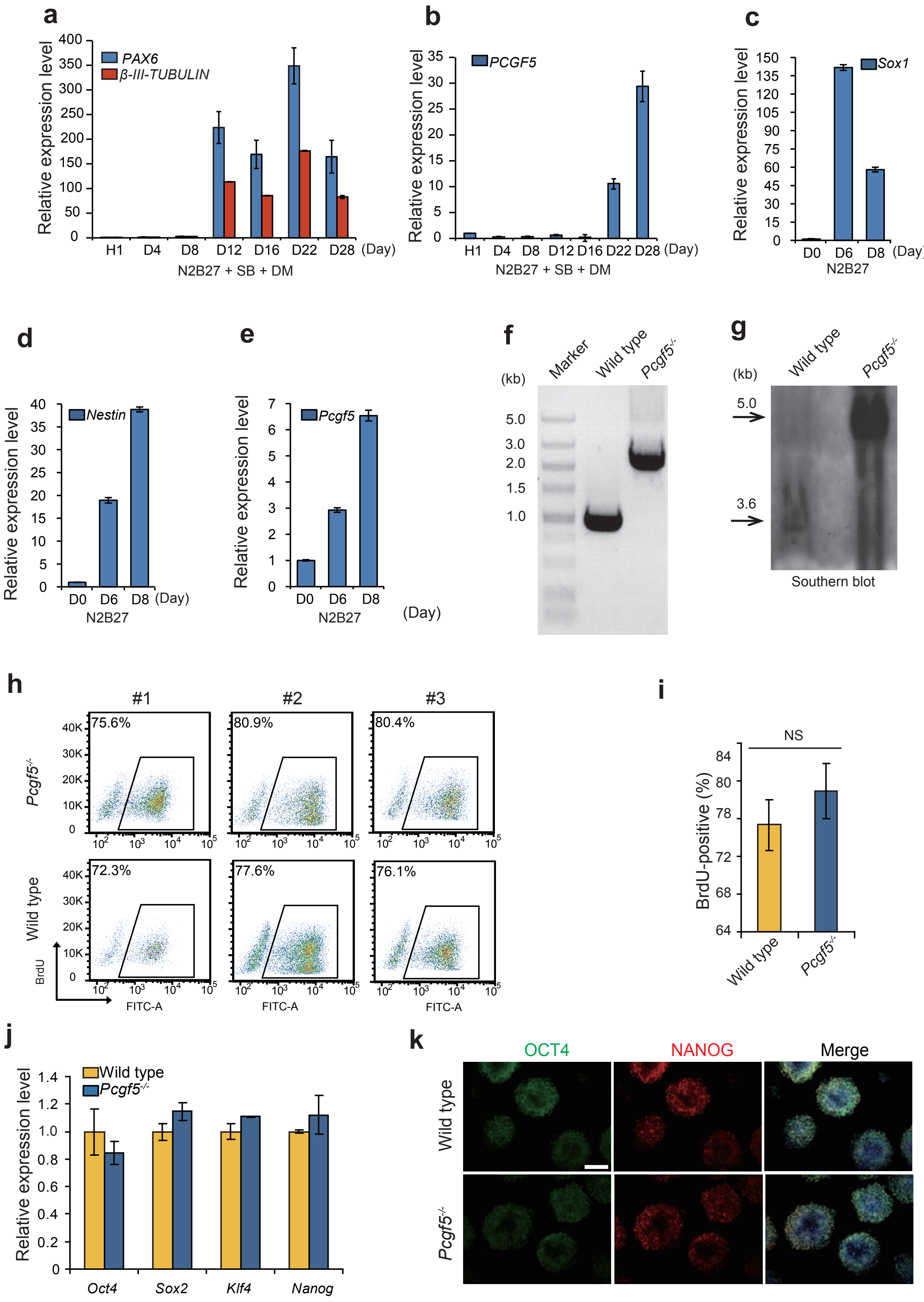

a
PAX6
β-III-TUBULIN
Relative expression level
H1 D4 D8 D12 D16 D22 D28 (Day)
N2B27 + SB + DM
b
PCGF5
c
Sox1
D0 D6 D8 (Day)
N2B27
d
Nestin
e
Pcgf5
f
Marker
Wild type
Pcgf5-/-
(kb)
5.0
3.0
2.0
1.5
1.0
g
3.6
Southern blot
h
#1
#2
#3
75.6%
80.9%
80.4%
72.3%
77.6%
76.1%
Wild type
BrdU
FITC-A
i
NS
BrdU-positive (%)
j
Wild type
Oct4
Sox2
Klf4
Nanog
k
OCT4
NANOG
Merge

Supplementary Figure 1 PCGF5 loss-of-function blocks mESC neural differentiation

a Gene expression analysis of *PAX6* and β*-III-TUBULIN* during neural differentiation of human embryonic stem cells (H1) from D0-D28 (n=3). Results are shown relative to H1 at day 0. **b** Gene expression analysis of *PCGF5* during neural differentiation of human embryonic stem cells (H1) from D0-D28 (n=3). Results are shown relative to H1 at day 0. **c** Gene expression analysis of *Sox1* during mESC neural differentiation from D0-D8 (n=3). Results are shown relative to day 0. **d** Gene expression analysis of *Nestin* during mESC neural differentiation from D0-D8 (n=3). Results are shown relative to day 0. **e** Gene expression analysis of *Pcgf5* during mESC neural differentiation from D0-D8 (n=3). Results are shown relative to day 0. **f** Gel shows PCR products amplified from genomic DNA of wild type and *Pcgf5*$^{-/-}$ mESCs. **g** Southern blot analysis of the targeted alleles. **h** Flow cytometry analysis of wild type and *Pcgf5*$^{-/-}$ mESCs. **i** Statistical analysis of BrdU-positive cells described in h. **j** Gene expression analysis of pluripotency genes in wild type and *Pcgf5*$^{-/-}$ mESCs (n=3). Results are shown relative to wild type. **k** Immunofluorescence analysis of NANOG and OCT4 in wild type and *Pcgf5*$^{-/-}$ mESCs. Scale bar represents 100 μm. Data in a-e, i, j, are represented as mean values ± s.d. with the indicated significance from Student's t-test (NS, no significant).

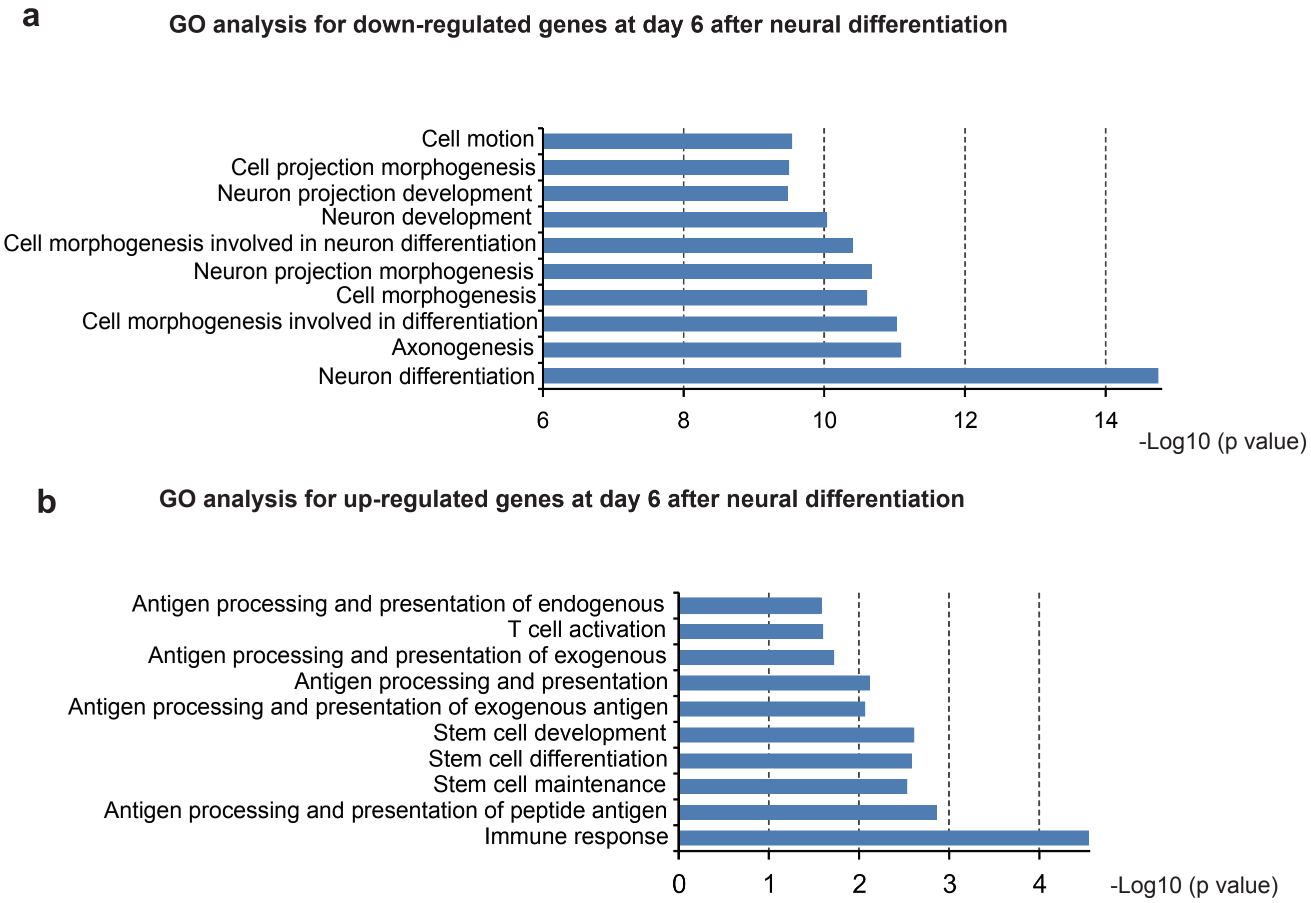
a
GO analysis for down-regulated genes at day 6 after neural differentiation
Cell motion
Cell projection morphogenesis
Neuron projection development
Neuron development
Cell morphogenesis involved in neuron differentiation
Neuron projection morphogenesis
Cell morphogenesis
Cell morphogenesis involved in differentiation
Axonogenesis
Neuron differentiation
6
8
10
12
14
-Log10 (p value)
b
GO analysis for up-regulated genes at day 6 after neural differentiation
Antigen processing and presentation of endogenous
T cell activation
Antigen processing and presentation of exogenous
Antigen processing and presentation
Antigen processing and presentation of exogenous antigen
Stem cell development
Stem cell differentiation
Stem cell maintenance
Antigen processing and presentation of peptide antigen
Immune response
0
1
2
3
4
-Log10 (p value)

Supplementary Figure 2 PCGF5 loss-of-function blocks neural differentiation

a Gene ontology (GO) analysis of differential expression of down-regulated genes in wild type and *Pcgf5*$^{-/-}$ mESCs at day 6 after neural differentiation. Results are expressed as –log10 (*p* value). **b** GO analysis of differential expression of up-regulated genes in wild type and *Pcgf5*$^{-/-}$ mESCs at day 6 after neural differentiation. Results are expressed as –log10 (*p* value).

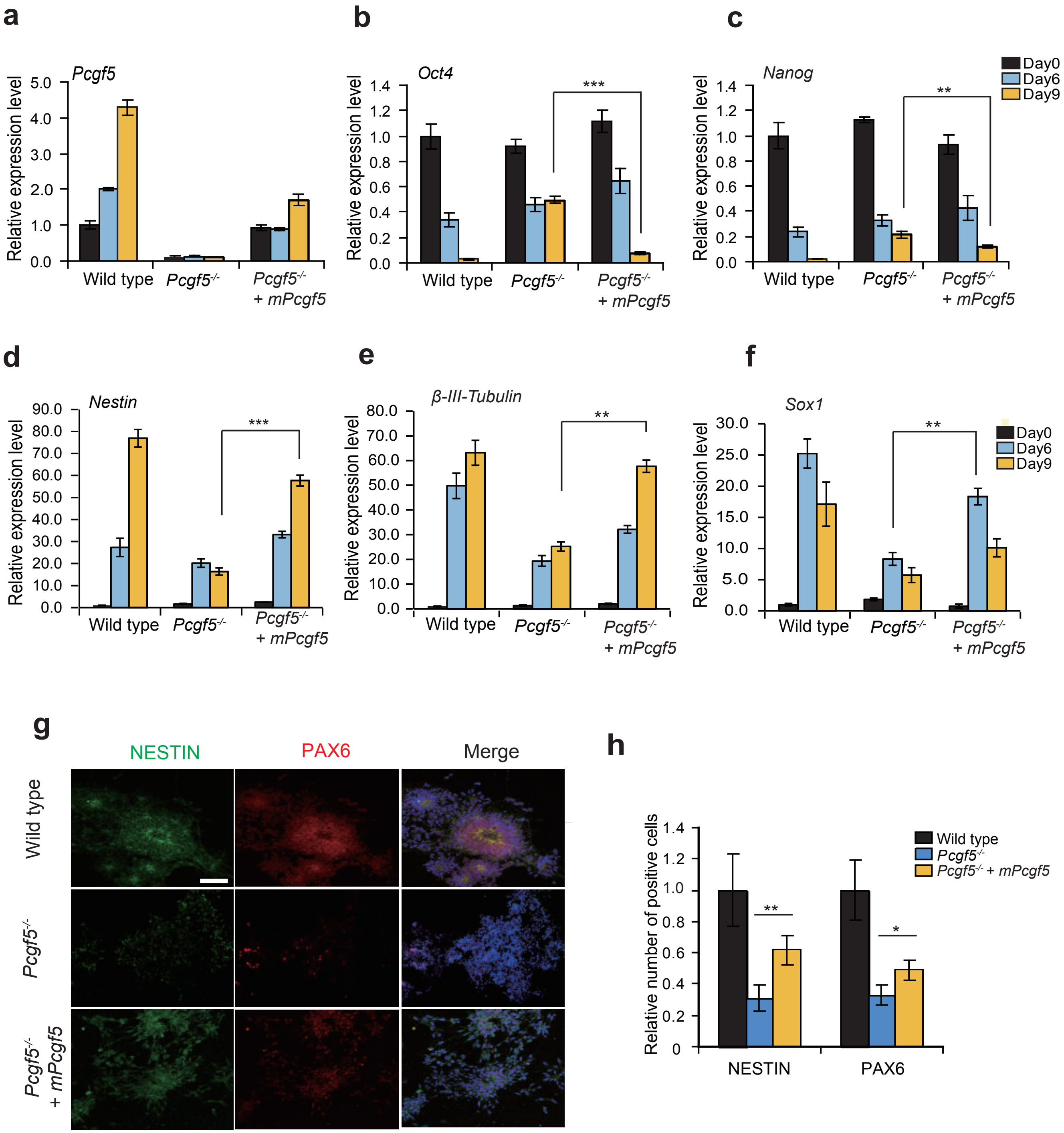

a
Pcgf5
Relative expression level
5.0
4.0
3.0
2.0
1.0
0.0
Wild type
Pcgf5-/-
Pcgf5-/- + mPcgf5
b
Oct4
1.4
1.2
1.0
0.8
0.6
0.4
0.2
0.0
***
c
Nanog
**
Day0
Day6
Day9
d
Nestin
90.0
80.0
70.0
60.0
50.0
40.0
30.0
20.0
10.0
0.0
***
e
β-III-Tubulin
**
f
Sox1
30.0
25.0
20.0
15.0
10.0
5.0
**
g
NESTIN
PAX6
Merge
Wild type
Pcgf5-/-
Pcgf5-/- + mPcgf5
h
Relative number of positive cells
Wild type
Pcgf5-/-
Pcgf5-/- + mPcgf5
**
*
NESTIN
PAX6

Supplementary Figure 3 Ectopic PCGF5 rescues mESC neural differentiation defects

a-c Gene expression analysis of *Pcgf5*, *Oct4* and *Nanog* in wild type, *Pcgf5*$^{-/-}$ and overexpression of *Pcgf5* in *Pcgf5*$^{-/-}$ mESCs during neural differentiation (n=3). Results are shown relative to wild type at day 0. **d-f** Gene expression analysis of *Nestin*, *β-III-tubulin*, *Sox1* in wild type, *Pcgf5*$^{-/-}$ and overexpression of *Pcgf5* in *Pcgf5*$^{-/-}$ mESCs during neural differentiation (n=3). Results are shown relative to wild type at day 0. **g** Immunostaining of the neural progenitor markers NESTIN and PAX6 in wild type, *Pcgf5*$^{-/-}$ and overexpression of *Pcgf5* in *Pcgf5*$^{-/-}$ mESCs at day 6 after neural differentiation. Scale bar represents 100 μm. **h** Statistical analysis of NESTIN and PAX6 positive cells in g (n=3). Data in a-f, h are represented as mean values ± s.d. with the indicated significance from Student's t-test (*p<0.05, **p<0.01, ***p<0.001).

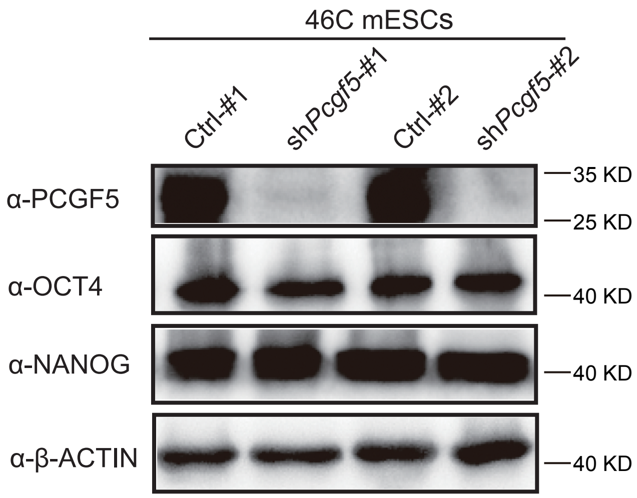

46C mESCs
Ctrl-#1
shPcgf5-#1
Ctrl-#2
shPcgf5-#2
α-PCGF5
α-OCT4
α-NANOG
α-β-ACTIN
35 KD
25 KD
40 KD
40 KD
40 KD

Supplementary Figure 4 SMAD2/TGF-β signaling pathway is activated in PCGF5-deficient NPCs

Western blot analysis of NANOG and OCT4 level in control and PCGF5-deficient 46C mESCs.

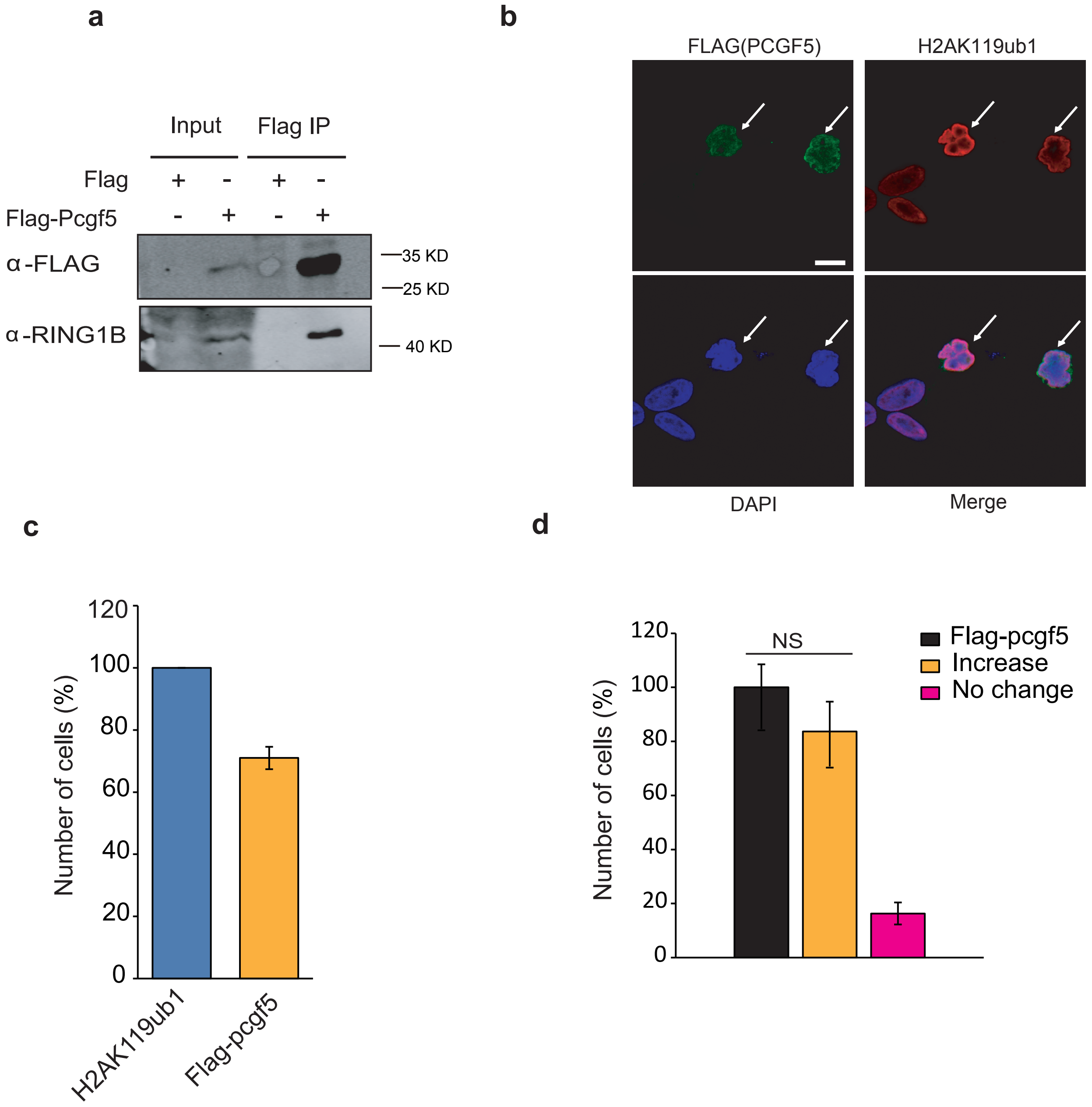
a
Input
Flag IP
Flag
+
-
+
-
Flag-Pcgf5
-
+
-
+
α-FLAG
α-RING1B
35 KD
25 KD
40 KD
b
FLAG(PCGF5)
H2AK119ub1
DAPI
Merge
c
Number of cells (%)
120
100
80
60
40
20
0
H2AK119ub1
Flag-pcgf5
d
NS
Flag-pcgf5
Increase
No change
Number of cells (%)
120
100
80
60
40
20
0

Supplementary Figure 5 PCGF5 increases histone H2AK119ub1 level *in vivo*

a Detection of the interaction between Flag-PCGF5 and RING1B. Flag-tagged empty vector was used as a control. **b** Immunostaining analysis of Flag-PCGF5 and histone H2AK119ub1 in 293T cells. Scale bar represents 100 μm. **c** Percentage of H2AK119ub1 and Flag-PCGF5 positive cells in b (n=3). **d** Immunostaining analysis of the percentage of cells with H2AK119ub1 in overexpressed Flag-PCGF5 in b and the correlation between Flag-PCGF5 positive cells and H2AK119ub1 level. Data in c, d are represented as mean values ± s.d. with the indicated significance from student's t-test (NS, no significance).

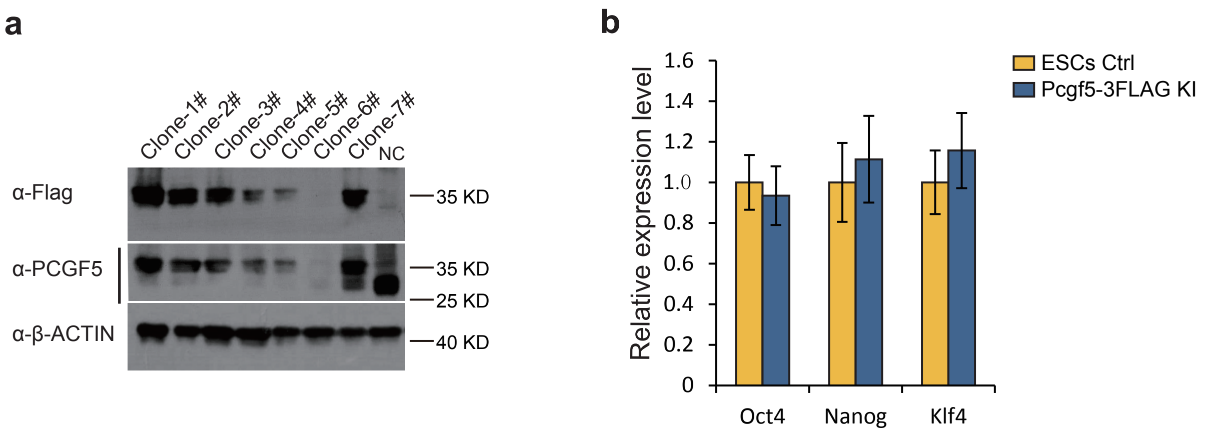

a
Clone-1#
Clone-2#
Clone-3#
Clone-4#
Clone-5#
Clone-6#
Clone-7#
NC
α-Flag
α-PCGF5
α-β-ACTIN
35 KD
35 KD
25 KD
40 KD
b
Relative expression level
1.6
1.4
1.2
1.0
0.8
0.6
0.4
0.2
0
Oct4
Nanog
Klf4
ESCs Ctrl
Pcgf5-3FLAG KI

Supplementary Figure 6 Effects of Flag-PCGF5 knockin on the expression of pluripotency genes in mESCs

a Western blot analysis of Flag (Flag-PCGF5) and PCGF5 in Flag-tagged PCGF5 knockin mESCs. **b** Gene expression analysis of pluripotency genes in wild type and Flag-PCGF5 knockin mESCs (n=3). Results are shown relative to wild type. Data in b are represented as mean values ± s.d..

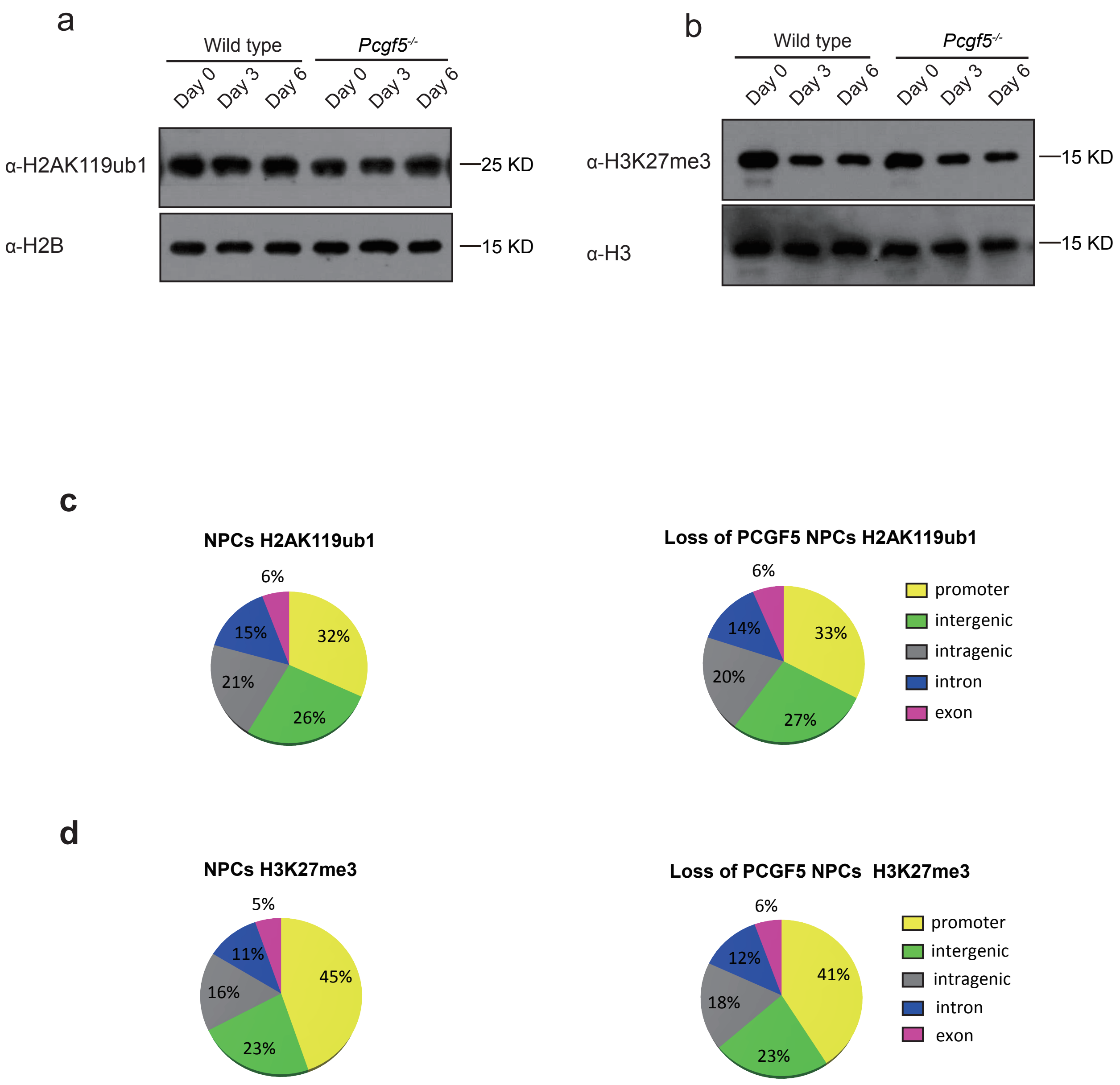

a
Wild type
Pcgf5-/-
Day 0
Day 3
Day 6
Day 0
Day 3
Day 6
α-H2AK119ub1
—25 KD
α-H2B
—15 KD
b
Wild type
Pcgf5-/-
Day 0
Day 3
Day 6
Day 0
Day 3
Day 6
α-H3K27me3
—15 KD
α-H3
—15 KD
c
NPCs H2AK119ub1
6%
15%
32%
21%
26%
Loss of PCGF5 NPCs H2AK119ub1
6%
14%
33%
20%
27%
promoter
intergenic
intragenic
intron
exon
d
NPCs H3K27me3
5%
11%
45%
16%
23%
Loss of PCGF5 NPCs H3K27me3
6%
12%
41%
18%
23%
promoter
intergenic
intragenic
intron
exon

Supplementary Figure 7 Effects of PCGF5 deficient on the level and distribution of H2AK119ub1 and H3K27me3

a Western blot analysis of histone H2AK119ub1 level during neural differentiation of both wild type and *Pcgf5*$^{-/-}$ mESCs. **b** Western blot analysis of histone H3K27me3 level during neural differentiation of both wild type and *Pcgf5*$^{-/-}$ mESCs. **c** Genome-wide distribution of histone H2AK119ub1 in both wild type and PCGF5-deficient NPCs at day 6 after neural differentiation. **d** Genome-wide distribution of histone H3K27me3 in both wild type and PCGF5-deficient NPCs at day 6 after neural differentiation.

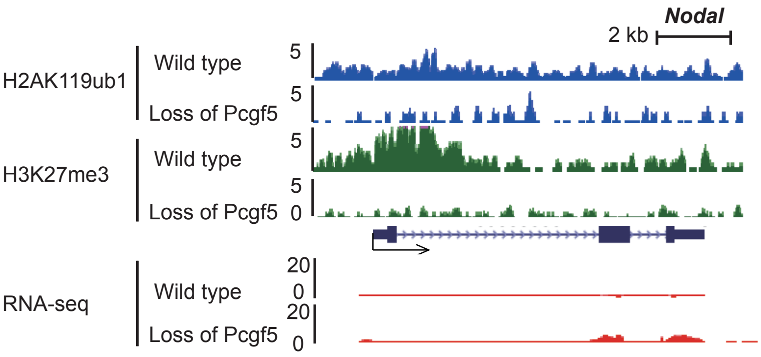

Nodal
2 kb
H2AK119ub1
Wild type
Loss of Pcgf5
H3K27me3
Wild type
Loss of Pcgf5
RNA-seq
Wild type
Loss of Pcgf5
5
5
5
5
0
20
0
20
0

Supplementary Figure 8 Gene track analysis of binding profiles at the Nodal promoter

UCSC genome browser view of H2AK119ub1 and H3K27me3 binding profiles at the promoter of *Nodal* gene in both wild type and PCGF5-deficient NPCs.

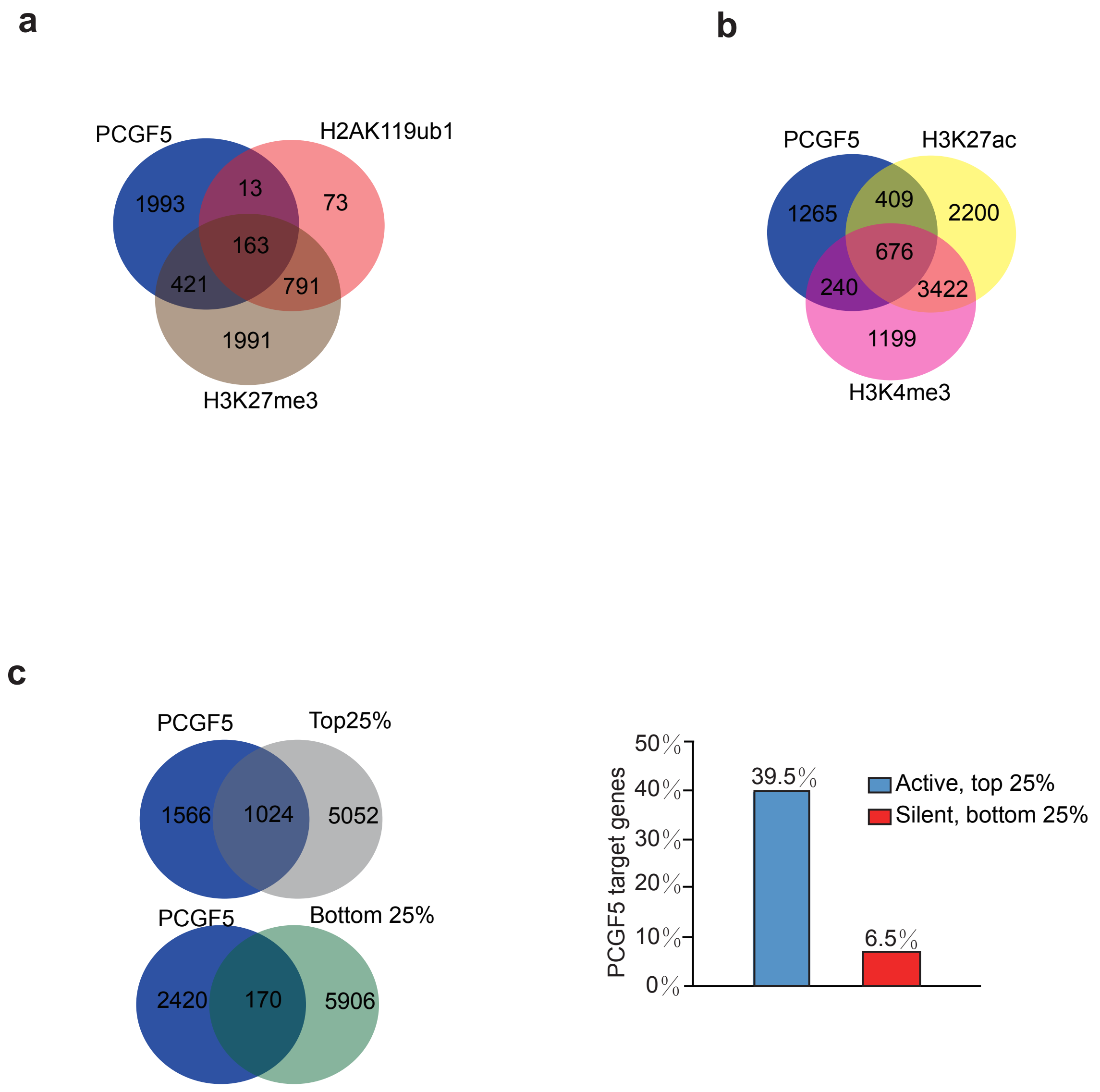
a
PCGF5
H2AK119ub1
1993
13
73
163
421
791
1991
H3K27me3
b
PCGF5
H3K27ac
1265
409
2200
676
240
3422
1199
H3K4me3
c
PCGF5
Top25%
1566
1024
5052
PCGF5
Bottom 25%
2420
170
5906
PCGF5 target genes
50%
40%
30%
20%
10%
0%
39.5%
6.5%
Active, top 25%
Silent, bottom 25%

Supplementary Figure 9 Overlap analysis of PCGF5 binding sites with histone repressive and active marks

a Venn diagram showing the overlap of genes bound by PCGF5 (blue), H2AK119ub1 (pink) and H3K27me3 (brown) in wild type NPCs at day 6 after neural differentiation. **b** Venn diagram showing the overlap of genes bound by PCGF5 (blue), H3K27ac (yellow) and H3K4me3 (purple) in wild type NPCs at day 6 after neural differentiation. **c** Percentage of PCGF5 target genes overlapped with the highest (top 25%, blue bar) and the lowest (bottom 25%, red bar) expressed genes in wild type NPCs at day 6 after neural differentiation.

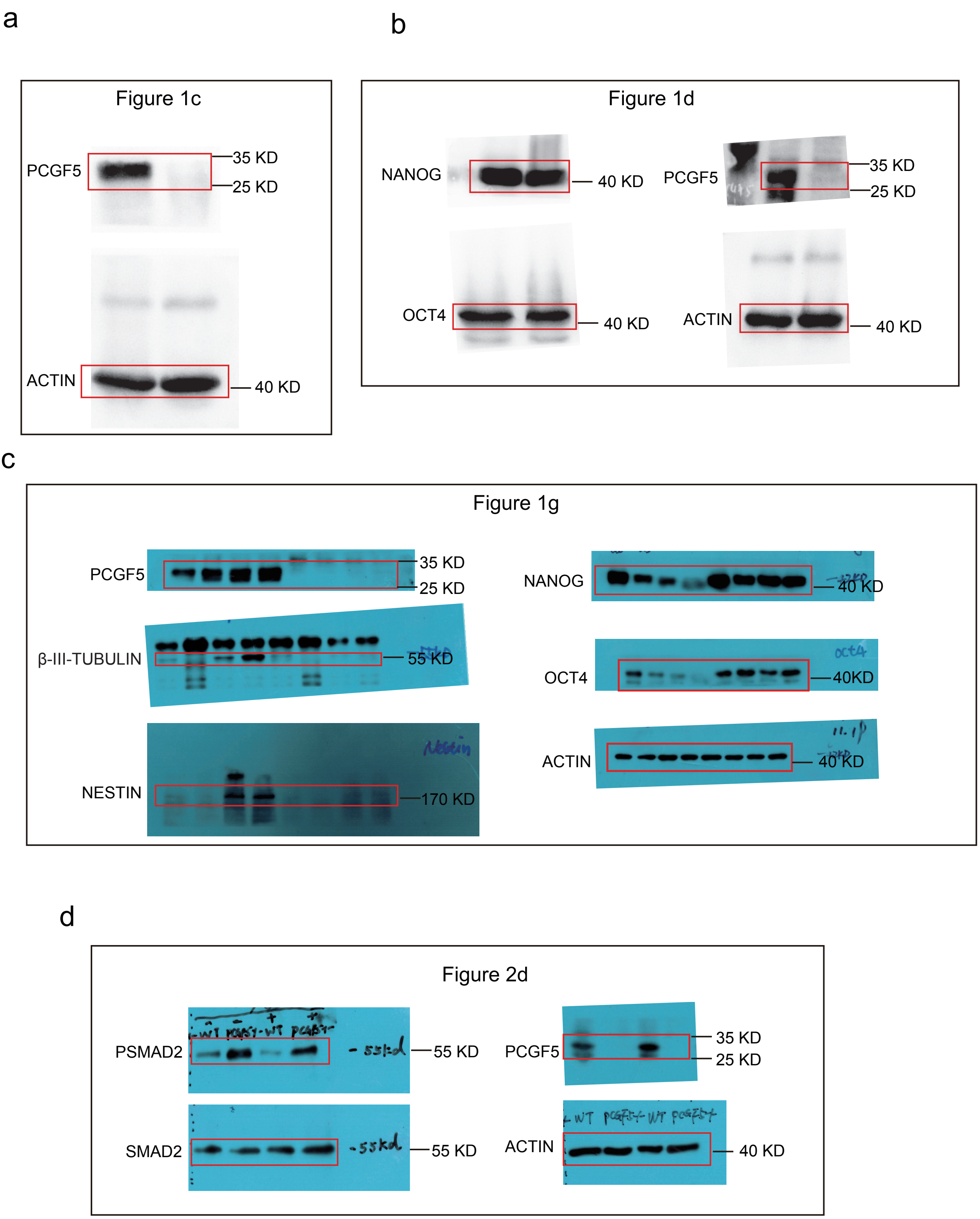
a
Figure 1c
PCGF5
35 KD
25 KD
ACTIN
40 KD
b
Figure 1d
NANOG
40 KD
PCGF5
35 KD
25 KD
OCT4
40 KD
ACTIN
40 KD
c
Figure 1g
PCGF5
35 KD
25 KD
β-III-TUBULIN
55 KD
NESTIN
170 KD
NANOG
40 KD
OCT4
40KD
ACTIN
40 KD
d
Figure 2d
PSMAD2
55 KD
SMAD2
55 KD
PCGF5
35 KD
25 KD
ACTIN
40 KD

Supplementary Figure 10 Original photos of Western blots are shown for figure 1 and 2

a Western blot analysis of PCGF5 expression in wild type and *Pcgf5*$^{-/-}$ mESCs for figure 1c. ACTIN was used as the loading control. Red box indicates the location of target protein. KD: Kilodaltons. **b** Western blot analysis of PCGF5, NANOG and OCT4 expressions in wild type and *Pcgf5*$^{-/-}$ mESCs for figure 1d. ACTIN was used as the loading control. Red box indicates the location of target protein. KD: Kilodaltons. **c** Western blot analysis of PCGF5, pluripotent markers (OCT4, NANOG), neural markers (NESTIN, β-III-TUBULIN) in wild type and *Pcgf5*$^{-/-}$ mESCs during neural differentiation of mESCs for figure 1g. ACTIN was used as the loading control. KD: Kilodaltons. **d** Western blot analysis of PSMAD2 in wild type and *Pcgf5*$^{-/-}$ cells at day 6 after neural differentiation for figure 2d. DMSO or LY2109761 (1 μM) was added during neural differentiation. ACTIN was used as the loading control. KD: Kilodaltons.

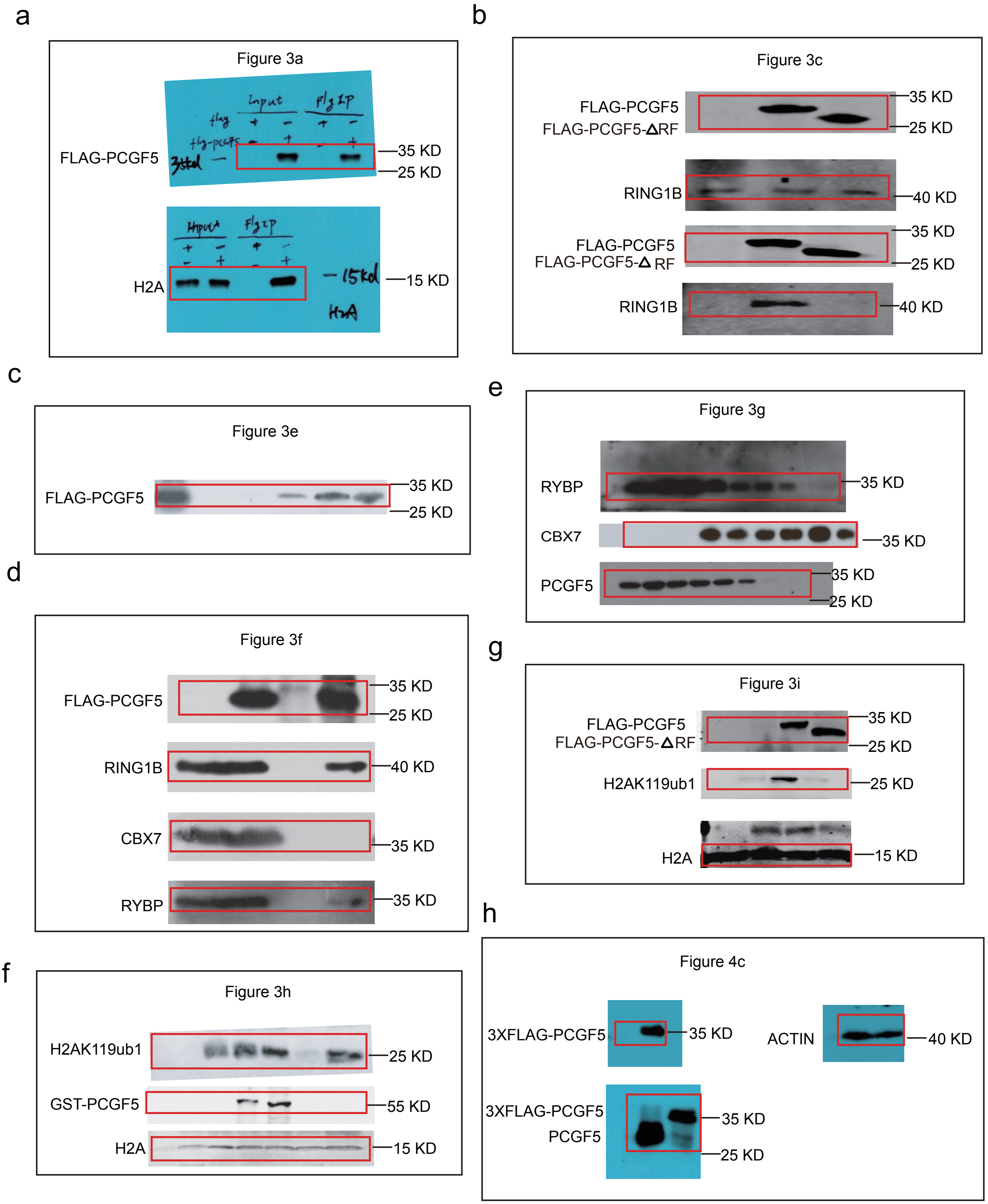
a
Figure 3a
FLAG-PCGF5
35 KD
25 KD
H2A
15 KD
b
Figure 3c
FLAG-PCGF5
FLAG-PCGF5-ΔRF
35 KD
25 KD
RING1B
40 KD
FLAG-PCGF5
FLAG-PCGF5-Δ RF
35 KD
25 KD
RING1B
40 KD
c
Figure 3e
FLAG-PCGF5
35 KD
25 KD
d
Figure 3f
FLAG-PCGF5
35 KD
25 KD
RING1B
40 KD
CBX7
35 KD
RYBP
35 KD
e
Figure 3g
RYBP
35 KD
CBX7
35 KD
PCGF5
35 KD
25 KD
g
Figure 3i
FLAG-PCGF5
FLAG-PCGF5-ΔRF
35 KD
25 KD
H2AK119ub1
25 KD
H2A
15 KD
f
Figure 3h
H2AK119ub1
25 KD
GST-PCGF5
55 KD
H2A
15 KD
h
Figure 4c
3XFLAG-PCGF5
35 KD
ACTIN
40 KD
3XFLAG-PCGF5
PCGF5
35 KD
25 KD

Supplementary Figure 11 Original photos of Western blots are shown for figures 3 and 4

a Western blot analysis of the interaction between Flag-PCGF5 and H2A for figure 3a. Flag-tagged empty vector was used as control. KD: Kilodaltons. **b** Western blot analysis of the interaction between RING1B and PCGF5 with or without a Ring-finger for figure 3c. KD: Kilodaltons. **c** GST pull-down assay using immobilized GST only or recombinant GST-Ring1b N terminus (GST-Ring1b-N), Ring finger (GST-Ring1b-RF), C terminus (GST-Ring1b-C), and full-length (GST-Ring1b) proteins probed with anti-FLAG antibody (Flag-PCGF5) by Western blot for figure 3e. KD: Kilodaltons. **d** Western blot analysis of the interaction between Flag-PCGF5 and PRC1 subunits by Flag co-IP in 293T cells for figure 3f. Flag-tagged empty vector was used as a control. KD: Kilodaltons. **e** Western blot analysis of every fraction from a 10%-30% sucrose gradient for figure 3g. KD: Kilodaltons. **f** Western blot analysis of *in vitro* H2A monoubquitinylation assay with nucleosome, GST purified PCGF5, PCGF4, RING1B, E1, E2 and ubiquitin for figure 3h. H2A as the loading control. KD: Kilodaltons. **g** Western blot analysis of *in vitro* H2A monoubquitinylation assay with purified Flag-tagged PCGF5 and Flag-tagged PCGF5 without a Ring-finger in 293T cells for figure 3i. H2A as the loading control. KD: Kilodaltons. **h** Western blot analysis of the Flag (Flag-PCGF5), PCGF5 in wild type and Pcgf5-3 × Flag knockin mESCs at day 6 after neural differentiation for figure 4c. ACTIN was used as the loading control. KD: Kilodaltons.

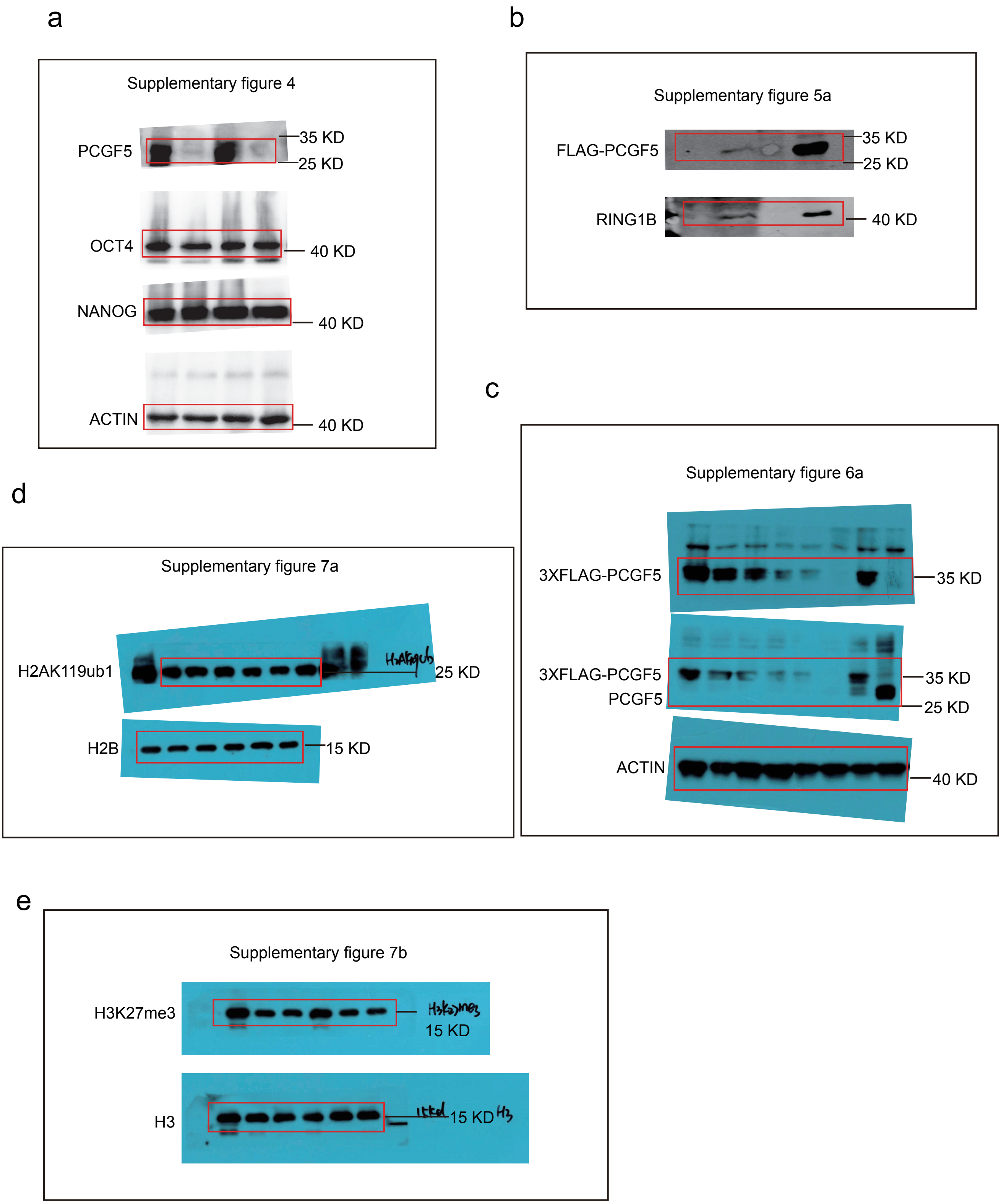

a
Supplementary figure 4
PCGF5
35 KD
25 KD
OCT4
40 KD
NANOG
40 KD
ACTIN
40 KD
b
Supplementary figure 5a
FLAG-PCGF5
35 KD
25 KD
RING1B
40 KD
c
Supplementary figure 6a
3XFLAG-PCGF5
35 KD
3XFLAG-PCGF5
PCGF5
35 KD
25 KD
ACTIN
40 KD
d
Supplementary figure 7a
H2AK119ub1
25 KD
H2B
15 KD
e
Supplementary figure 7b
H3K27me3
15 KD
H3
15 KD

Supplementary Figure 12 Original photos of Western blots are shown for Supplementary figure 4-7

a Western blot analysis of NANOG and OCT4 levels in control and PCGF5-deficient 46C mESCs for supplementary figure 4. ACTIN was used as the loading control. KD: Kilodaltons. **b** Western blot analysis of the interaction between RING1B and PCGF5 for supplementary figure 5a. KD: Kilodaltons. **c** Western blot analysis of Flag (Flag-PCGF5) and PCGF5 in Flag-tagged PCGF5 knockin mESCs for supplementary figure 6a. ACTIN was used as the loading control. KD: Kilodaltons. **d** Western blot analysis of histone H2AK119ub1 levels during neural differentiation of both wild type and *Pcgf5*$^{-/-}$ mESCs for supplementary figure 7a. H2B was used as the loading control. KD: Kilodaltons. **e** Western blot analysis of histone H3K27me3 levels during neural differentiation of both wild type and *Pcgf5*$^{-/-}$ mESCs for supplementary figure 7b. H3 was used as the loading control. KD: Kilodaltons.

Supplementary Table 1 Sequences of Oligos, related to experimental procedures

Sequences of shRNA Oligos, related to experimental procedures	
	Oligonucleotieds sequences(5`-3`)
pLKO-sh*pcgf5*-#1	CCGG GTTGGATGTGCTGTGCAAT CTCGAG ATTGCACAGCACATCCAAC TTTTTG
	AATTCAAAAA GTTGGATGTGCTGTGCAAT CTCGAG ATTGCACAGCACATCCAAC
pLKO-sh*pcgf5*-#2	CCGG GCAGTATCGACCGAGAATT CTCGAG AATTCTCGGTCGATACTGC TTTTTG
	AATTCAAAAA GCAGTATCGACCGAGAATT CTCGAG AATTCTCGGTCGATACTGC
Sequences of TALEN designed primers, Related to experimental procedures	
	Oligonucleotieds sequences(5`-3`)
PGK-NEO-F（P1）	cgg ggtacc AATTCTACCGGGTAGGGGAGGCGCT
PGK-NEO-R（P2）	cgc ggatcc CCCCAGCTGGTTCTTTCCGCCT
PGK-PURO-F（P1）	cgg ggtacc TACCGGGTAGGGGAGGC
PGK-PURO-R（P2）	cgc ggatcc CAGACATGATAAGATACATTGATGAG
m*Pcgf5*-talen-R-F（P3）	CGC GGATCC CGACAGTGACGGAATGCCT
m*Pcgf5*-talen-R-R（P4）	CCC AAGCTT ACTTTTCGTACATATAAAAATTAGGATAAT
m*Pcgf5*-talen-L-F（P5）	CG GAATTC CAAACTGGGAGTGGTGGGTC
m*Pcgf5*-talen-L-R（P6）	CGG GGTACC GCTTGATCAGATAGCCTTTACAG
Sequences of CRISPR/Cas9 designed primers for 3XFlag tagged PCGF5 mES stable cell lines, Related to experimental procedures	
	Oligonucleotieds sequences(5`-3`)
Cas9-pcgf5-sgRNA-F	ACAAAGGTATGCGTAAAGCT
Cas9-pcgf5-sgRNA-R	AGCTTTACGCATACCTTTGT

Supplementary Table 2 List of primers used for qRT-PCR analysis

Application	Species	Gene	Forward primer	Reverse primer
qRT-PCR	Mouse	*Gapdh*	AACTTTGGCATTGTGGAAGGGCTCA	TTGGCAGCACCAGTGGATGCAGGGA
qRT-PCR	Mouse	*Klf4*	AACATGCCCGGACTTACAAA	TTCAAGGGAATCCTGGTCTTC
qRT-PCR	Mouse	*Lefty1*	CCAACCGCACTGCCCTTAT	CGCGAAACGAACCAACTTGT
qRT-PCR	Mouse	*Lefty2*	CAGCCAGAATTTTCGAGAGGT	CAGTGCGATTGGAGCCATC
qRT-PCR	Mouse	*Nanog*	CTCAAGTCCTGAGGCTGACA	TGAAACCTGTCCTTGAGTGC
qRT-PCR	Mouse	*Nestin*	CCCTGAAGTCGAGGAGCTG	CTGCTGCACCTCTAAGCGA
qRT-PCR	Mouse	*Nodal*	TTCAAGCCTGTTGGGCTCTAC	TCCGGTCACGTCCACATCTT
qRT-PCR	Mouse	*Oct4*	TAGGTGAGCCGTCTTTCCAC	GCTTAGCCAGGTTCGAGGAT
qRT-PCR	Mouse	*Pcgf5*	ATGGCTACCCAAAGGAAACA	AACTTGGTTGCCACACCTTG
qRT-PCR	Mouse	*Sox1*	GCACACAGCGTTTTCTCGG	ACATCCGACTCCTCTTCCC
qRT-PCR	Mouse	*Sox2*	CTGCAGTACAACTCCATGACCAG	GGACTTGACCACAGAGCCCAT
qRT-PCR	Mouse	*β-III-tubulin*	ACTTGGAACCTGGAACCATGG	GGCCTGAATAGGTGTCCAAAGG
qRT-PCR	Human	*GAPDH*	TCCAAAATCAAGTGGGGCGAT	TTCTAGACGGCAGGTCAGGTC
qRT-PCR	Human	*KDM2A*	CTGCTCCCTCTGGGTAGTCT	TCTGCCTCTGGGAAAATCCG
qRT-PCR	Human	*KDM2B*	GTCGGATGAGCACTCGAAGA	CCAGGTTTGAGCCGCTTGC
qRT-PCR	Human	*KDM3A*	TGAGCCACACAGACAGGTTG	TTGAAGATGGTGGTGTCGCA
qRT-PCR	Human	*KDM3B*	TTCGCGGACACTGCGG	AAAGATCGCTAGGTCCTGGG
qRT-PCR	Human	*KDM4A*	AAAGCAGTAGGATCGGCCAG	AATCTACAGCCCAAAGCCCC
qRT-PCR	Human	*KDM4B*	GCAGGTCTCACCGGAAACG	TACCCTCCCCAGGGAACTTG
qRT-PCR	Human	*KDM4C*	AAAGTTACATCCCCTCCGCC	CTAAACACGATGCTGGCTGC
qRT-PCR	Human	*KDM4D*	TCCTTCATTCCGGTACTGCG	GGAAGCAGGAGTCGTTGGAT
qRT-PCR	Human	*KDM5A*	GGGGTTTCTGTGTTGAACGG	GGGGAACCGGTGGAGAAAAG
qRT-PCR	Human	*KDM5B*	AAGATGGGGTTTGCTCCTGG	GTCTGTGGTCAGGTTTGGCT
qRT-PCR	Human	*KDM5C*	CATCACCAGTCAGTGGAGCA	TCGGGGGAGGATCAGAATGT
qRT-PCR	Human	*KDM5D*	AGGAATACAAGCCCCACAGC	TGTAGGCTCTGGATCAGGCT
qRT-PCR	Human	*KDM6A*	AGCTGAAGGAAAAGTGGAGTCT	AGGCAGCATTCTTCCAGTAGT
qRT-PCR	Human	*KDM6B*	GTACCGCACTGAGGAGCTG	GGGCTCAGGGAAAGGCG
qRT-PCR	Human	*JMJD5*	GCGCGGGTTTTATACTCTGC	TGCATTTCTCGCGGCTCATA
qRT-PCR	Human	*FIH1*	CGACTAGGCCCATTCCGC	CCACAGGCTCCTCATTCTCAA
qRT-PCR	Human	*JMJD1C*	GCATTACATCACGACGCAGG	GGACTATTTGCTTGGGCACG
qRT-PCR	Human	*JARID2*	ATGGTGTATTTTGGAAGCTC	CTGGAACCATTGAAAACATG
qRT-PCR	Human	*UTY*	GCACCACTGGTTTTGTAGCTG	GCACTGTGTCCAGTTGCTTG
qRT-PCR	Human	*WDFY3*	GTGTGCAGTCTGTGATATCTGAA	ACTTAAGTACCTGGACTATTGCAT
qRT-PCR	Human	*TET2*	GGGAAGCCAGAATAGTCGTG	ACCCCGAAGTTACGTCTTTC
qRT-PCR	Human	*PHF2*	CTGCCTCTAACCACAGCGAG	GGTGAGTGTGGCGTAGATCC
qRT-PCR	Human	*PHF8*	AAGGTTCAACGTCCCTGCTC	CCCCTGTGAAGGAAGCTCTG
qRT-PCR	Human	*HSPBAP1*	TAAAGCGTTTTCCTCAGTTCCG	AGGTGATCCTCTTCCTGTCCT
qRT-PCR	Human	*PCGF1*	ATCGCGATGAGGCTTCGGAA	GACACTCTGTGATGGTGGTG
qRT-PCR	Human	*PCGF2*	ATGCATCGGACTACACGGAT	CGTCACACATGGGGCAGTAT
qRT-PCR	Human	*PCGF3*	ACCGAGTGTCTGCACACCTT	CATTTCCGCTTCTTGGAGGC
qRT-PCR	Human	*PCGF4*	GAGCTAAATCCCCACCTGAT	AGTAGTGGTCTGGTCTTGTG
qRT-PCR	Human	*PCGF5*	GCAGGTGGGACAGACTTGG	CCTTTTGGTGGTTCCGCTTT
qRT-PCR	Human	*PCGF6*	GACGAGGAGTTGGAAGAAGA	GAGATTAATCAGGCGCTCCT
qRT-PCR	Human	*PAX6*	ATGCAGAACAGTCACAGCGG	GTTGGACACCTGCAGAATTC
qRT-PCR	Human	*β-III-TUBULIN*	ATGGACAGTGTCCGCTCAGG	TTTCACACTCCTTCCGCACC

Supplementary Table 3 List of antibodies used in this study

Name of antibody	WB	ChIP-qPCR	ChIP-Seq	IF	Company	Catalog Number
Flag	1:1000	2μg	-	1:200	Sigma	F1804
H2AK119ub1	1:1000	2μg	5μg	1:1000	Cell Signaling Technology	#8240
H3K27me3	1:1000	-	-	-	GeneTex	GTX54106
H3	1:5000	-	-	-	Abcam	ab1791
H3K27me3	-	2μg	5μg	-	Abcam	ab6002
H2A	1:1000	-	-	-	Abcam	ab18255
H2B	1:1000	-	-	-	Cell Signaling Technology	12364S
Anti-FLAG M2 Magnetic Beads	-	-	15μL	-	Sigma Aldrich	M8823
Mouse IgG	-	2μg	-	-	Santa Cruz Biotechnology	sc-2025
Nanog	1:5000	-	-	1:500	Novus Life Sciences	58842
Nestin	1:500	-	-	1:500	BD Biosciences	611658
Oct3/4	1:2000	-	-	1:200	Santa Cruz Biotechnology	sc-5279
Pax6	-	-	-	1:100	Abcam	ab5790
Pcgf5	1:200	-	-	-	Abcam	ab201511
pSmad2	1:2000	-	-	-	Cell Signaling Technology	#3108
Rabbit IgG	-	2μg	-	-	Santa Cruz Biotechnology	sc-2027
β-III-tubulin	1:1000	-	-	-	Cell Signaling Technology	#5568S
Ring1B	1:1000	-	-	-	Abcam	ab194666
Rybp	1:1000	-	-	-	Abcam	ab5976
Cbx7	1:1000	-	-	-	Abcam	ab21873
Smad2/3	1:2000	-	-	-	Cell Signaling Technology	#3102
β-actin	1:5000	-	-	-	Abcam	ab3280
Abbreviations: WB, Western Blotting; ChIP, Chromatin immunoprecipitation; IF, Immunofluorescence						

Supplementary Table 4 List of primers used for ChIP-qPCR analysis

Application	Species	Gene	Forward primer	Reverse primer
ChIP-qPCR	Mouse	*Cdh2*	GTAGGAACATCCCTGGTCGG	CGAGTTCTTTTGCCACTCCG
ChIP-qPCR	Mouse	*Lefty1*	CTTTTGCACACCTGGGAAGC	TCCCAGTGTCTGGAATGTGC
ChIP-qPCR	Mouse	*Lefty2*	GGGCACTTTTAGGGACGCAT	AGGACCTGTTCCTCGGTCAT
ChIP-qPCR	Mouse	*Nestin*	GAGGAACCCAGCCTGCTATG	CAAGAGTCGCCTCGAGAAGG
ChIP-qPCR	Mouse	*Nodal*	TCCAAACAGCCCACCATGAG	GGTGGAGTAGAGCCCAACAG
ChIP-qPCR	Mouse	*Pou3f2*	CGGGAGGGGTCATCCTTTTC	TCAAATGCCCTAAGCCCTCG
ChIP-qPCR	Mouse	*Sox1*	AAGTTGCGGTCCCAGAGTTG	CTCCTCTTGTCGGCTCGAAG
