# Supplementary material for: PCGF5 is required for neural differentiation of embryonic stem cells
Source: Nat Commun. 2018 May 15;9:1463. doi: 10.1038/s41467-018-03781-0 (PMC5954019; doi:10.1038/s41467-018-03781-0)
Supplement: Supplementary file 3 — Description of Additional Supplementary Files [file 41467_2018_3781_MOESM3_ESM.pdf]

Description of Additional Supplementary Files

File Name: Supplementary Data 1
Description: Differentially expressed genes in WT vs. PCGF5-/- ESCs at Day 0 and Day 6.

File Name: Supplementary Data 2
Description: PCGF5 binding profiles in ESCs and NPCs.

File Name: Supplementary Data 3
Description: H2AK119ub1 and H3K27me3 profiles in WT and PCGF5-/- ESCs and NPCs.
